# Reduction of hyperoxic acute lung injury in mice by Formononetin

**DOI:** 10.1371/journal.pone.0245050

**Published:** 2021-01-07

**Authors:** Yin Chen, Dong Wei, Jin Zhao, Xiangnan Xu, Jingyu Chen

**Affiliations:** 1 Department of Thoracic Surgery, Wuxi People’s Hospital, Nanjing Medical University, Wuxi, Jiangsu, China; 2 Department of Thoracic Surgery, Shanghai General Hospital, Shanghai Jiaotong University, Shanghai, China; Ohio State University, UNITED STATES

## Abstract

**Background:**

The antioxidant and anti-inflammatory features of Formononetin, an isoflavone constituent extracted from traditional Chinese medicine, have been reported. The present study investigated that whether Formononetin plays a benefit on hyperoxic ALI.

**Methods:**

C57BL/6 mice were exposed to hyperoxia for 72 h to produce experimental hyperoxic ALI model. Formononetin or vehicle was administrated intraperitoneally. Samples from the lung were collected at 72 h post hyperoxia exposure for further study. Pulmonary microvascular endothelial cells isolated from the lung of C57BL/6 mice were used for *in vitro* study.

**Results:**

Formononetin pretreatment notably attenuated hyperoxia-induced elevating pulmonary water content, upregulation of proinflammatory cytokine levels and increasing infiltration of neutrophil in the lung. Western blot analyses showed that Formononetin enhanced the expression of nuclear factor erythroid-2-related factor 2 (Nrf2) which is a key transcription factor regulating the expression of heme oxygenase-1 (HO-1). Formononetin increased HO-1 expression and activity compared with vehicle-treated animals. Moreover, Formononetin reversed hyperoxia-caused the reduction of M2 macrophage polarization. However, pretreatment of a HO-1 inhibitor reduced the protective effect of Formononetin on hyperoxic ALI. Cell study showed that the Formononetin-induced upregulation of HO-1 was abolished when the Nrf2 was silenced.

**Conclusions:**

Formononetin pretreatment reduces hyperoxia-induced ALI via Nrf2/HO-1-mediated antioxidant and anti-inflammatory effects.

## Introduction

The mechanism of acute respiratory distress syndrome (ARDS) is complicated [1–3]. Oxidative stress is believed to play a vital role in the pathogenesis of ARDS, especially in hyperoxia-induced acute lung injury (ALI) [1, 4]. Reduction of oxidants such as reactive oxygen species (ROS) by induction of antioxidants is believed to be an important strategy for attenuation of ARDS [5]. Nuclear factor erythroid 2-related factor 2 (Nrf2) is a key transcription factor regulating the expression of antioxidants such as heme oxygenase-1 (HO-1) [4]. Evidence has shown that upregulation of Nrf2/HO-1 by pharmacological agents protected the lung in ALI animal model [6]. Nrf2/HO-1 is a logical therapeutic target for hyperoxic ALI.

Formononetin is an isoflavone constituent extracted from traditional Chinese medicine such as *Radix Astragali* [7]. Formononetin has a range of pharmacological features including anti-inflammatory and antioxidant properties. Evidence has shown that Formononetin protected cortical neurons via suppressing inflammatory reaction [8]. NLRP3 inflammasome signaling pathway was inhibited by Formononetin in dextran sulfate sodium-induced acute colitis animal models [9]. Cisplatin-induced intracellular ROS accumulation in LLC-PK1 cells was inhibited by Formononetin [10]. Formononetin increased HO-1 and down-regulated the protein expression of BACH1 in a traumatic brain injury animal model [11]. Moreover, the elevation of HO-1 by Formononetin was reported in animal models of high-fat diet-induced neuroinflammation [12], Methotrexate-induced kidney injury [13], and ovalbumin-induced allergic asthma [14]. Formononetin activated AMP-activated protein kinase in high-fat diet-induced obesity and bone loss in mice [15]. Acetaminophen-induced hepatotoxicity was dampened by Formononetin via elevating Nrf2 activity [16]. Evidence has shown that Formononetin dampened LPS-induced ALI in mice [17]. However, the effect of Formononetin on hyperoxic ALI is unclear and the potential antioxidant mechanism of Formononetin is still needed to be elucidated. The present study was conducted to test the hypothesis that Formononetin reduces hyperoxic ALI.

## Materials and methods

### Animals

All the animals used in the present study were cared in full compliance with the National Institutes of Health guidelines for the use of experimental animals. We obtained the approval for use of animals in the present research from the Institutional Animal Care and Use Committee of Nanjing Medical University (IACUC 14030359. 201803).

C57BL/6 mice were obtained from Laboratory Animal Center of Nanjing Medical University. All the mice used in the present research were 8−12-week-old male mice.

### Hyperoxic ALI

Before the experiment, mice were housed in airtight and individually ventilated cages on a 12-h light: dark cycle for at least 1 week to habituate the environment. The mice were allowed standard chow and water *ad libitum*. Mice were assigned to control (room air+Vehicle) and other four groups (room air+Formononetin, hyperoxia+Vehicle, hyperoxia+Formononetin, and zinc protoporphyrin (ZnPP, an inhibitor of HO-1)+hyperoxia+Formononetin) (n = 8). To induce hyperoxic ALI, the mice were housed in the airtight cages and exposed to hyperoxia (> 95% oxygen) for 72 h as previously described [18]. Room air exposure was served as control. The animals were observed continuously. To minimize mice suffering and distress, all efforts were performed. Euthanasia with CO_2_ asphyxiation was performed for animals that suffered going on to die irreversibly (such as becoming comatose or losing >20% body weight).

### Formononetin administration

Formononetin (10 or 100 mg/kg, Aladdin Bio-Chem Technology company, Shanghai, China) [9] was administrated intraperitoneally 1 hour before hyperoxia exposure, and followed by additional dosage per 24 h. Formononetin was dissolved in dimethyl sulfoxide (DMSO) as described previously [9]. Vehicle (equivalent DMSO) was injected at same time points. To inhibit the activity of HO-1, ZnPP (5 mg/kg, St. Louis, MO, USA) was administered intraperitoneally 30 min before Formononetin injection [19] and followed by additional dosage per 24 h.

The mice were euthanized at 72 h after hyperoxia exposure by CO_2_ asphyxiation. The level of Evan’s blue (EB) dye-labeled albumin infiltrated in the lung was an indicator of pulmonary microvascular permeability. At 30 min prior to sacrifice, EB dye-labeled albumin (50 μg/g) was injected via tail vein [20]. The EB dye-labeled albumin level was measured spectrophotometrically. Samples were collected and stored at -80°C for further investigation.

### Lung edema assay

The fresh harvested lung tissue was weighted (wet weight) immediate and then was dried in an oven at 80°C for 48 h (dry weight). The wet to dry (W/D) weight ratio of the lung tissue was calculated to evaluate the pulmonary edema.

### Collection of bronchoalveolar lavage fluid (BALF)

The lung was harvested and a tube was inserted into the airway. Phosphate buffered saline was instilled and aspirated via the tube (1mL for three times) to collect BALF. The collected BALF was centrifuged (1000×g) for 10 min. A hemocytometer was used to measure cell counts in BALF.

### Cytokine assay

Enzyme linked immunosorbent assay (ELISA) kits was used to detect the levels of cytokines in lung tissues, including interleukin (IL)-1β and IL-6 (R&D Systems, Minneapolis, MN, USA), according to the manufacturer’s instructions.

### Histological assay

The lung was obtained and fixed with formalin immediately. Then, the lung tissues were washed, dehydrated, and embedded in paraffin. Hematoxylin and eosin were used to stain the lung tissue sections. The sections were evaluated by a light microscopy. The pathological severity of the lung was evaluated following a previously described method in a blinded manner [21]. Briefly, the following four parameters were scored independently to evaluate the lung tissue pathological severity: congestion, hemorrhage, infiltration of white blood cell, and alveolar septa thickness.

### Measurement of malondialdehyde (MDA) activity

MDA activity in lung tissues was measured by an assay kit of MDA according to the manufacturer’s instructions (Nanjing Jincheng Bioengineering Institute, Nanjing, China).

### Measurement of antioxidant enzyme activity

A superoxide dismutase (SOD) assay kit was used to measure the SOD activity in the lung according to the manufacturer’s instructions (Nanjing Jiancheng Bioengineering Institute, Nanjing, China).

HO-1 degradates heme and generates biliverdin. Thus, the generated biliverdin is an indicator of HO-1 activity. We measured the HO-1 activity as previously described [22].

### Western blotting analysis

The protein concentrations were determined by BCA protein assay kit (Thermo Fisher Scientific, Asheville, NC, USA). Samples (containing 50 μg protein) were separated on polyacrylamide-sodium dodecyl sulfate (SDS) gel and then transferred to nitrocellulose membrane. The Membranes were blocked with fat-free milk then probed with primary (Nrf2, dilution 1:500; Santa Cruz Biotechnology, Dallas, TX, USA; Lamin B, dilution 1:500; Santa Cruz Biotechnology, Dallas, TX, USA; HO-1, Abcam, Cambridge, MA, USA; β-actin, dilution 1:500; Santa Cruz Biotechnology, Dallas, TX, USA) and secondary antibodies (dilution 1:5,000; Santa Cruz Biotechnology). Image J software was used to analyze the bands.

### Cell study

We isolated pulmonary microvascular endothelial cells (PMVECs) from the lung of C57BL/6 mice as previously described [23]. We cultured the isolated PMVECs in Dulbecco's modified Eagle's medium at 37°C with 5% CO_2_. Briefly, cultured PMVECs were seeded into 96-well plates (100μL/well) at a density of 2×10^6^ cells/well. The cultured PMVECs were treated with or without Formononetin (10 to 160 μM) for 20 h. Then, MTT solution (10 μL) was added and incubated for 4 h to determine the viability of PMVECs. Then, DMSO (150μL) was added to dissolve generated formazan. A microplate reader was used to measure the cell viability at an absorbance of 570 nm.

Small interfering RNA technique (siRNA) was used to silence Nrf2 (siRNA-Nrf2, Santa Cruz Biotechnology, Santa Cruz, CA, USA) in PMVECs with Lipofectamine 2000 according to the manufacturer's instructions (Active Motif, Carlsbad, CA, USA). Negative control siRNA was obtained from Santa Cruz Biotechnology (Santa Cruz, CA, USA).

### Real-time reverse transcriptase-polymerase chain reaction analysis

Trizol reagent (Invitrogen Life Technologies, Carlsbad, CA, USA) was used to extract total RNA. A spectrophotometer (Thermo Fisher, Boston, MA, USA) was used to measure the extracted RNA concentration (300–500 ng/μL). A SuperScript III cDNA kit (Invitrogen Life Technologies, Carlsbad, CA, USA) was used to generate cDNA from extracted total RNA. Then, real-time polymerase chain reaction was performed with PrimeScript™ RT reagent kit. The primers for HO-1 were 5´-AAGCCGAGAATGCTGAGTTCA-3´ (forward), and 5´-GCCGTGTAGATATGGTACAAGGA-3´ (reverse), respectively (PrimerBank ID: 6754212a1). A housekeeping gene, β-actin, was used as internal control. The primers for β-actin were 5´-GGCTGTATTCCCCTCCATCG-3´ (forward), and 5´-CCAGTTGGTAACAATGCCATGT-3´ (reverse), respectively (PrimerBank ID: 6671509a1). The primers for chemokine (C-X-C motif) ligand (CXCL) 10 were 5´-CCAGAATCGAAGGCCATCAA-3´ (forward), and 5´-CATTTCCTTGCTAACTGCTTTCAG-3´ (reverse), respectively [24]. The primers for monocyte chemoattractant protein (MCP) 1 were 5´- CATCCACGTGTTGGCTCA-3´ (forward), and 5´- GATCATCTTGCTGGTGAATGAGT-3´ (reverse), respectively [24]. The primers for IL-1β were 5´-TGTAATGAAAGACGGCACACC-3´ (forward), and 5´-TCTTCTTTGGGTATTGCTTGG-3´ (reverse), respectively [24]. The primers for arginase (Arg)-1 were 5´-GAATGGAAGAGTCAGTGTGG-3´ (forward), and 5´-AATGACACATAGGTCAGGGT-3´ (reverse), respectively [24]. The primers for CD163 were 5´-GACGACAGATTCAGCGACTT-3´ (forward), and 5´-CCGAGGATTTCAGCAAGTCCA-3´ (reverse), respectively [24].

### Nrf2 activity analysis

A TransAM™ Nrf2 kit was used to detect the Nrf2 binding activity to antioxidant response elements according to the manufacturer’s instructions (Active Motif, Carlsbad, CA, USA).

### Statistical analysis

All data are represented as means±SD. The data were analyzed with one-way analysis of variance followed by Bonferroni *t*-test (SPSS 20, IBM, Armonk, NY, USA). Differences in values of *P* < 0.05 were considered significant.

## Results

### Effect of Formononetin on Nrf2 and HO-1 *in* *vivo*

**Fig 1:**
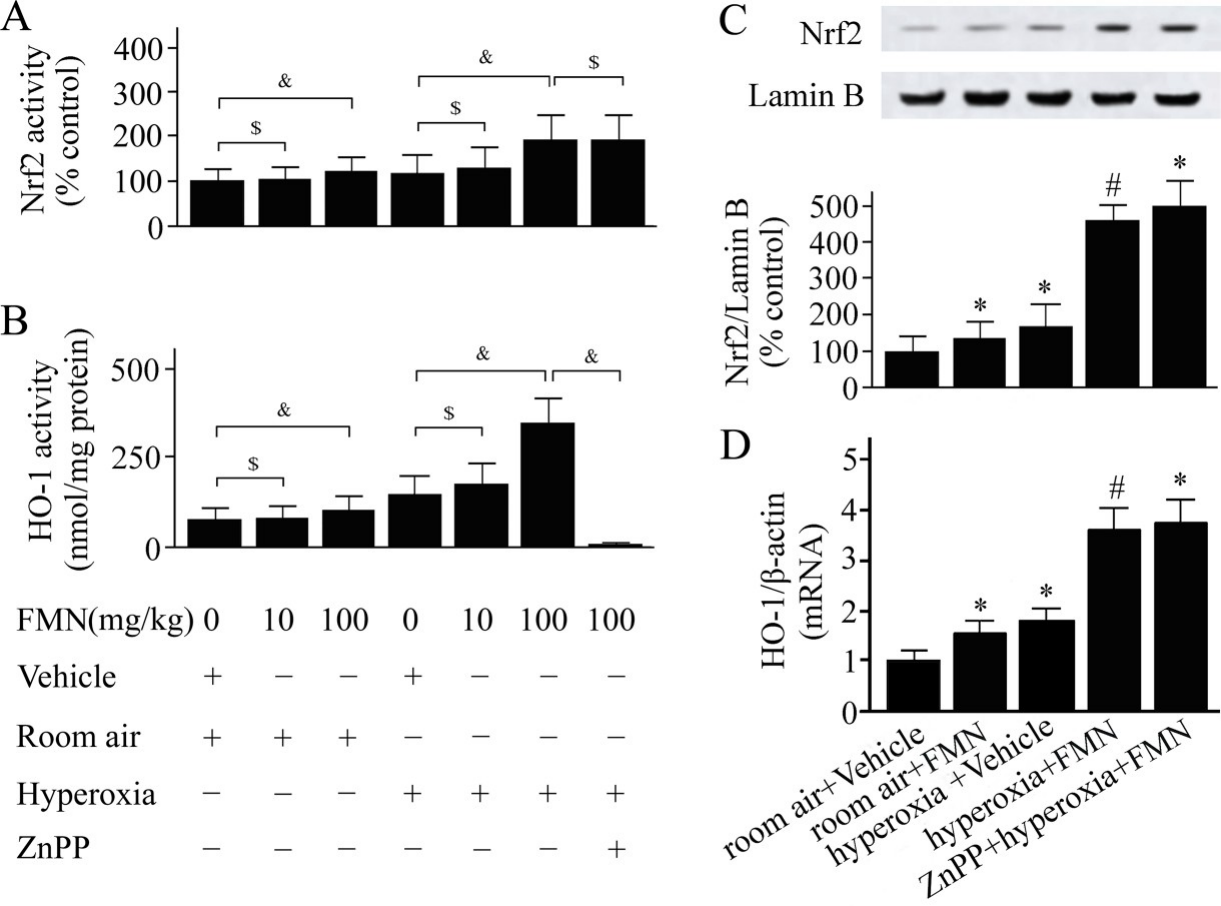
C57BL/6 mice were pretreated with Formononetin (FMN) or vehicle and exposed to hyperoxia or room air. Nuclear factor erythroid 2-related factor 2 (Nrf2) activity (A), heme oxygenase (HO)-1 activity (B), levels of Nrf2 in the nucleus (C), and HO-1 mRNA levels (D) were measured at 72 h after hyperoxia or room air exposure. Data are presented as mean ± SD. ^*&*^*P* < 0.05; ^*$*^*P* > 0.05; *P < 0.05 compared with vehicle-treated mice exposed to room air; ^#^*P* < 0.05 compared with vehicle-treated mice exposed to hyperoxia.

**Fig 2:**
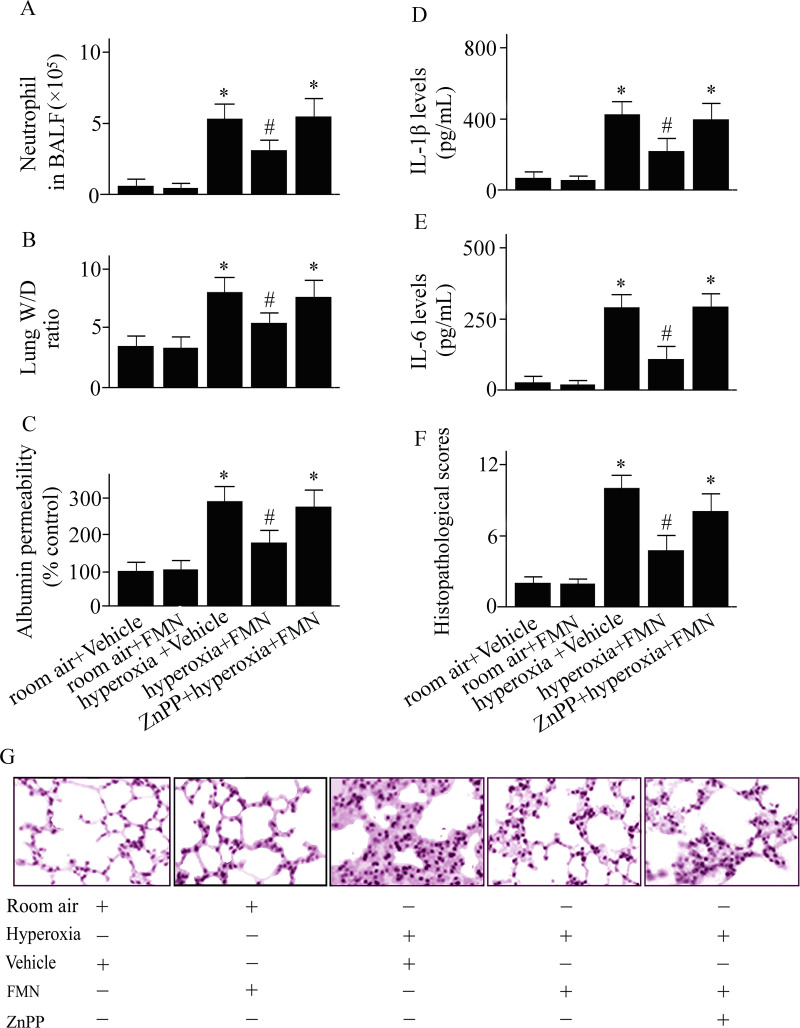
C57BL/6 mice were pretreated with Formononetin (FMN) or vehicle and exposed to hyperoxia or room air. Numbers of neutrophil cells in bronchoalveolar lavage fluid (A), the wet to dry (W/D) weight ratio of lung tissues (B), pulmonary microvascular albumin permeability (C), interleukin (IL)-1β (D) and IL-6 (E) levels in lung tissues, pulmonary histopathological score (F), and lung tissue sections (G; original magnifications: ×100) were measured at 72 h after hyperoxia or room air exposure. Data are presented as mean ± SD. *P < 0.05 compared with vehicle-treated mice exposed to room air; ^#^*P* < 0.05 compared with vehicle-treated mice exposed to hyperoxia.

As shown in Fig 1A, Formononetin 100- but not 10- mg/kg enhanced Nrf2 activation in mice exposed to hyperoxia. Similarly, the HO-1 activity was elevated by 100 mg/kg of Formononetin treatment (Fig 2B). We used 100 mg/kg of Formononetin in the following study. As shown in Fig 1C, Formononetin increased Nrf2 levels. The level of HO-1 mRNA was significantly increased in Formononetin-treated mice compared with vehicle-treated mice (Fig 1D). The threshold cycles of HO-1 and β-actin in the room air+Vehicle group were 25.27 and 15.28, respectively; and in the hyperoxia+Formononetin group were 23.39 and 15.24, respectively.

### Effect of Formononetin on hyperoxia-induced ALI

Pulmonary inflammation and edema, features of ALI, were investigated after hyperoxia challenge in vehicle-treated and Formononetin-treated mice. As shown in Fig 1, a significant increase in neutrophil infiltration (Fig 2A), the W/D weight ratio (Fig 2B), pulmonary microvascular permeability (Fig 2C), IL-1β (Fig 2D) and IL-6 (Fig 2E) concentrations in lungs were observed in vehicle-treated mice at 72 h after hyperoxic exposure. Hyperoxic exposure also causes pulmonary histopathological changes (Fig 1F and 1G). These hyperoxia-induced lung inflammation and edema were significantly dampened in Formononetin-treated versus vehicle-treated mice (Fig 2). To examine the role of Formononetin-induced elevating of HO-1 in hyperoxic ALI, we pretreated the mice with a HO-1 inhibitor. The protective effect of Formononetin on hyperoxic ALI was notably inhibited in the mice that pretreated with a HO-1 inhibitor (Fig 2). The data suggest that the induction of HO-1 played a vital role in the protective effect of Formononetin on hyperoxic ALI.

### Effect of Formononetin on MDA and antioxidant enzyme activity

**Fig 3:**
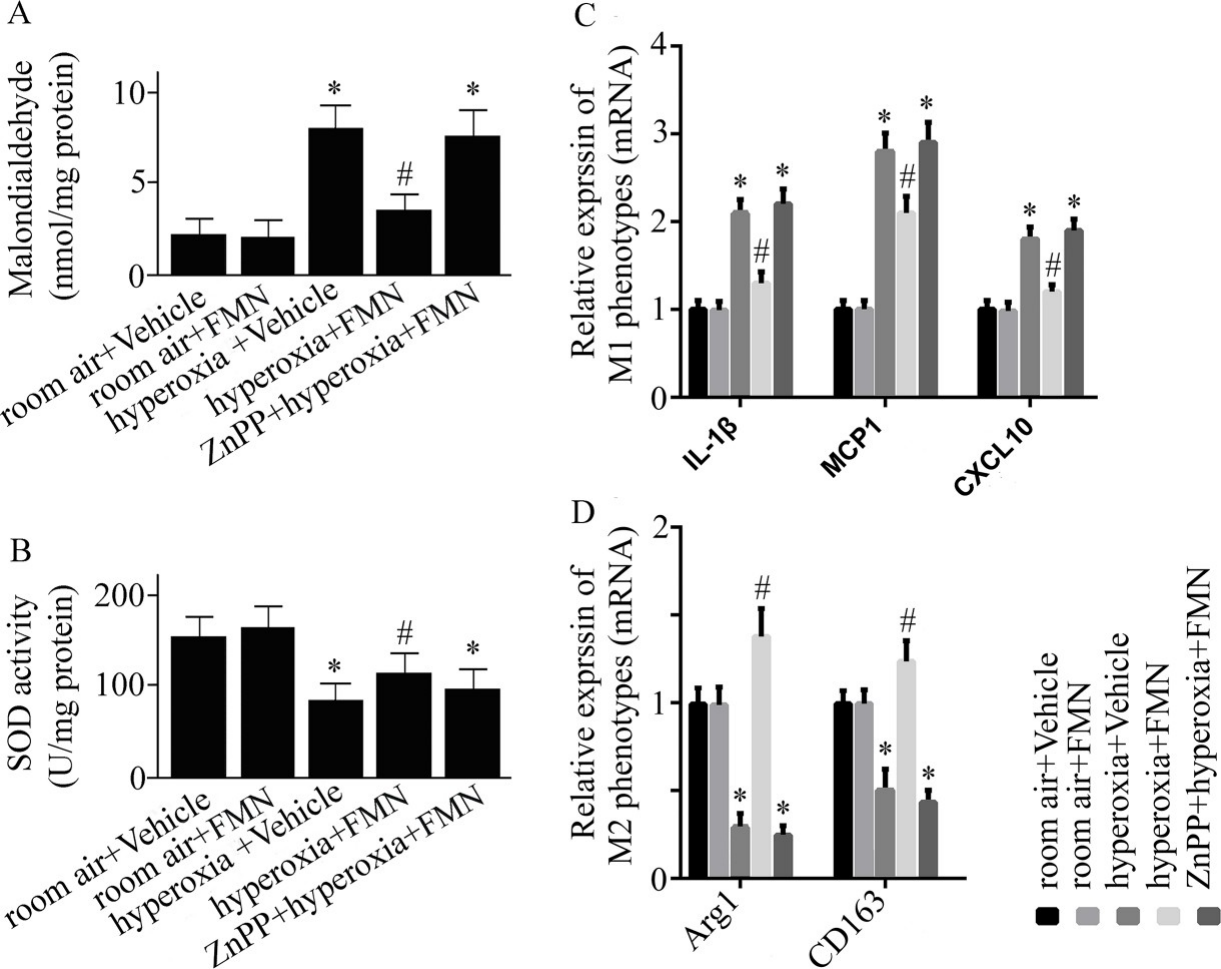
C57BL/6 mice were pretreated with Formononetin (FMN) or vehicle and exposed to hyperoxia or room air. Malondialdehyde (MDA) activity (A), superoxide dismutase (SOD) activity (B), relative mRNA expression of interleukin (IL)-1β, monocyte chemoattractant protein (MCP) 1, (C-X-C motif) ligand (CXCL) 10 (C), and relative expression of arginase (Arg)-1 as well as CD163 mRNA (D) were measured at 72 h after hyperoxia or room air exposure. Data are presented as mean ± SD. *P < 0.05 compared with vehicle-treated mice exposed to room air; ^#^*P* < 0.05 compared with vehicle-treated mice exposed to hyperoxia.

We measured the MDA activity to detect the oxidant levels. The MDA activity in lungs was increased markedly in mice exposed to hyperoxia compared with mice exposed to room air (Fig 3A). The hyperoxia-induced elevation of MDA was reduced in Formononetin-treated versus vehicle-treated mice (Fig 3A).

The activity of SOD was markedly reduced in vehicle-treated mice exposed to hyperoxia (Fig 3B). Formononetin treatment reversed the reduction of SOD activity (Fig 3B).

### Effect of Formononetin on macrophage polarization

Hyperoxia exposure caused an elevated mRNA expression of M1 phenotypes (IL-1β, MCP1, CXCL10), and a reduced M2 phenotypes (Arg1 and CD163) mRNA expression (Fig 3C and 3D). Formononetin reversed the hyperoxia-caused changes (Fig 3C and 3D).

### Effect of Formononetin on Nrf2 and HO-1 *in* *vitro*

**Fig 4:**
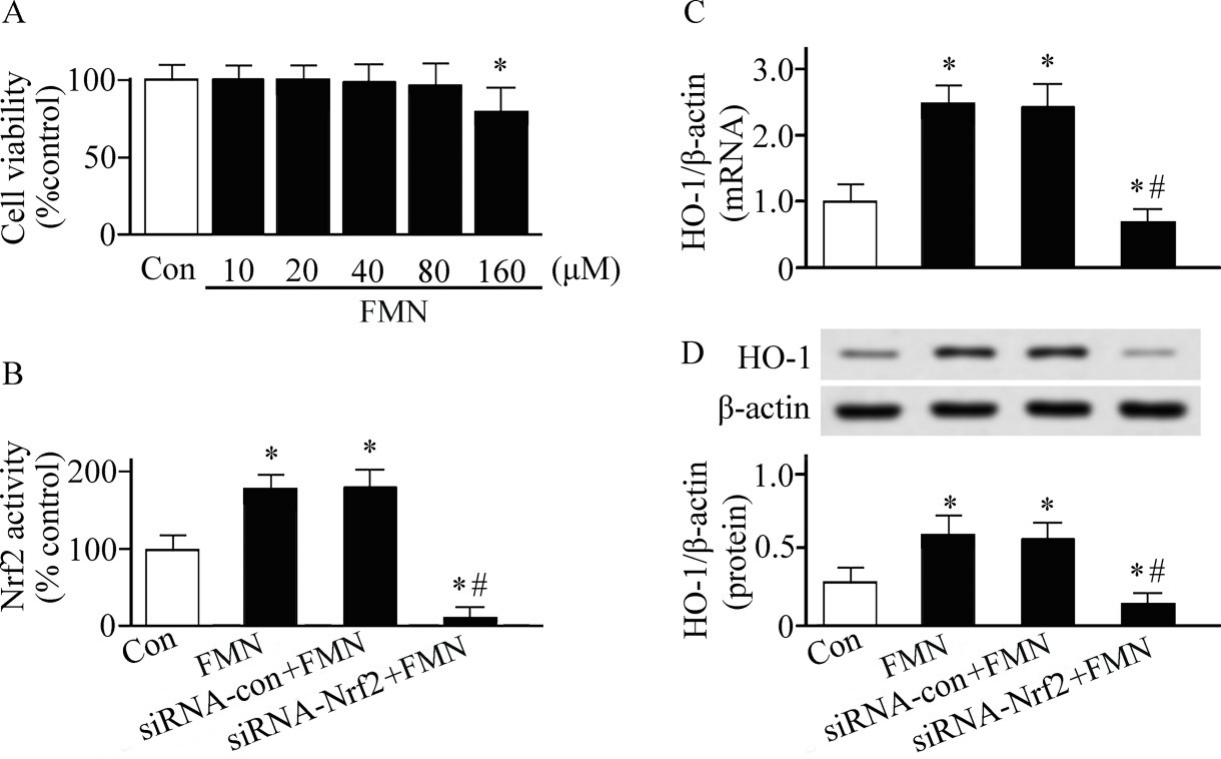
Pulmonary microvascular endothelial cells were cultured in Dulbecco's modified Eagle's medium and treated with or without Formononetin (FMN). The cell studies were performed in triplicate. (A) Cell viability was detected by MTT assay. (B) Nuclear factor erythroid 2-related factor 2 (Nrf2) DNA-bingding activity was detected by a TransAM™ Nrf2 kit. Heme oxygenase (HO)-1 expression was measured by real-time reverse transcriptase-polymerase chain reaction (C) and western blotting analysis (D). *P < 0.05. Con, control group. siRNA, small interfering RNA.

MTT assay was performed to investigate the influence of Formononetin on cell viability (Fig 4A). As shown in Fig 4, Formononetin activated Nrf2 and increased HO-1 expression in PMVECs. To determine why Formononetin can induce HO-1, the upstream signal of HO-1 was examined. The Nrf2 was silenced in PMVECs by using siRNA technique. We found that the Formononetin-induced HO-1 expression was inhibited in Nrf2 silenced PMVECs (Fig 4C and 4D). This result suggests that Nrf2 is the upstream signal of Formononetin-induced HO-1.

## Discussion

The antioxidant feature of Formononetin has been reported in a number of preclinical animal experiments and cell studies [10, 11, 16, 25]. However, the underlying antioxidant mechanism of Formononetin has not been fully characterized. The present study using a hyperoxia-induced ALI animal model showed that Formononetin reduced hyperoxia-induced ALI. Moreover, our data suggest that the protective effects of Formononetin on the lung are associated with upregulation of HO-1, as the protective effect of Formononetin was abolished when pretreated with a HO-1 inhibitor. Furthermore, we examined the upstream signal of Formononetin-induced HO-1 *in vitro*. We found that Nrf2 is the upstream signal of Formononetin-induced HO-1, since the Formononetin-induced elevating of HO-1 was markedly inhibited when the Nrf2 was silenced. These data suggest that Formononetin exerts its antioxidant effect via activation of Nrf2/HO-1 pathway.

Antioxidant enzymes, such as SOD and HO-1, degenerate oxidants [4, 26]. Excessive generated oxidants such as ROS play a deleterious role in the pathogenesis of ARDS. Inhibition of ROS generation by Formononetin has been reported in oxygen glucose deprivation/reoxygenation-induced cardiomyocytes injury [25]. However, the antioxidant mechanism of Formononetin has not been fully characterized. HO-1 is an inducible antioxidant enzyme and is suggested as an important endogenous antioxidant source [22, 27]. Evidence has shown that HO-1 is involved in the defense mechanism of the lung [27]. HO-1 gene deficiency mice experienced enhanced pulmonary inflammation and injury compared with wild-type mice [28]. Exogenous HO-1 gene transfer reduced susceptibility to hyperoxia-induced ALI in toll-like receptor 4 knockout mice [29]. Induction of HO-1 helped reduction of hyperoxia-induced lung inflammation and cell death [30]. Medicinal plants that may induce HO-1 have shown their benefits on ALI [6, 31]. Formononetin has shown its ability on upregulation of HO-1 in a traumatic brain injury animal model [11]. Evidence showed that Formononetin can enhance HO-1 in high-fat diet-induced neuroinflammation in mice [12]. In a rat model of methotrexate-stimulated kidney injury, Formononetin significantly up-regulated HO-1 and inhibited oxidative stress [13]. Moreover, improved ovalbumin-induced airway inflammation and oxidative stress, and elevated HO-1 expression were detected in Formononetin-treated animals [14]. Our data corroborate this finding. The expression and activity of HO-1 was markedly enhanced in Formononetin-treated mice compared with vehicle-treated animals that exposed to hyperoxia. SOD is one of the endogenous antioxidant enzymes. The current results showed that the activity of SOD was partly reversed by Formononetin treatment. As the HO-1 but not SOD was notably induced by Formononetin, we next examined whether HO-1 plays a vital role in the protective effect of Formononetin on hyperoxic ALI. A specific HO-1 inhibitor, ZnPP, was administrated to inhibit the HO-1. As expected, the protective effect of Formononetin on hyperoxic ALI was markedly inhibited when the HO-1 was inhibited. Our preclinical animal studies suggest that the beneficial effect of Formononetin on hyperoxic ALI could come from activation of HO-1.

Nrf2, an important transcription factor, plays a vital role in the regulation of HO-1 expression [4]. Induction of Nrf2/HO-1 pathway has been reported to play a benefit in preclinical animal studies [31, 32]. Post-liver transplantation ALI in rats was reduced by hemin via induction of HO-1 [31]. Evidence has shown that Nrf2/HO-1 axis played protective effects in hyperoxic ALI [27]. Formononetin has shown its ability on activation of Nrf2 in acetaminophen-induced hepatotoxicity in mice [16]. Our preclinical animal studies showed that the activation of Nrf2/HO-1 pathway was enhanced in Formononetin-treated mice exposed to hyperoxia for 72 h. Furthermore, we performed in vitro experiments using cultured PMVECs. We found that Nrf2 played a central role in Formononetin-induced elevation of HO-1, since the effect of Formononetin on the induction of HO-1 was abolished when Nrf2 was silenced.

Macrophages play an important role in the pathogenesis of ALI [33]. M1 phenotypes of macrophages are known as pro-inflammatory macrophages; while, M2 phenotypes have anti-inflammatory roles. During ALI, M1 macrophage polarization was enhanced resulting in the release of pro-inflammatory cytokines and inflammatory cell infiltration. Evidence showed that the reduction of M2 polarized macrophages contributed to the ALI [34]. HO-1 has shown its role on the induction of M2 macrophage polarization [35, 36]. Our results showed that hyperoxia-stimulated the reduction of M2 macrophages was reversed by Formononetin. However, this effect was abolished when the HO-1 was inhibited. The present data suggested that Formononetin can induce M2 macrophage polarization through a HO-1-dependent manner. Formononetin may reduce ALI, partly, via HO-1-mediated inducting M2 macrophage polarization.

## Conclusions

Formononetin pretreatment reduces hyperoxia-induced ALI via Nrf2/HO-1-mediated antioxidant and anti-inflammatory effects.

## Supporting information

S1 Fig Original images of Fig 2D. (TIF)

S2 Fig Original images of Fig 3D. (TIF)
