# Supplementary figures and images for: Reduction of hyperoxic acute lung injury in mice by Formononetin
Source: PLoS One. 2021 Jan 7;16(1):e0245050. doi: 10.1371/journal.pone.0245050 (PMC7790402; doi:10.1371/journal.pone.0245050)

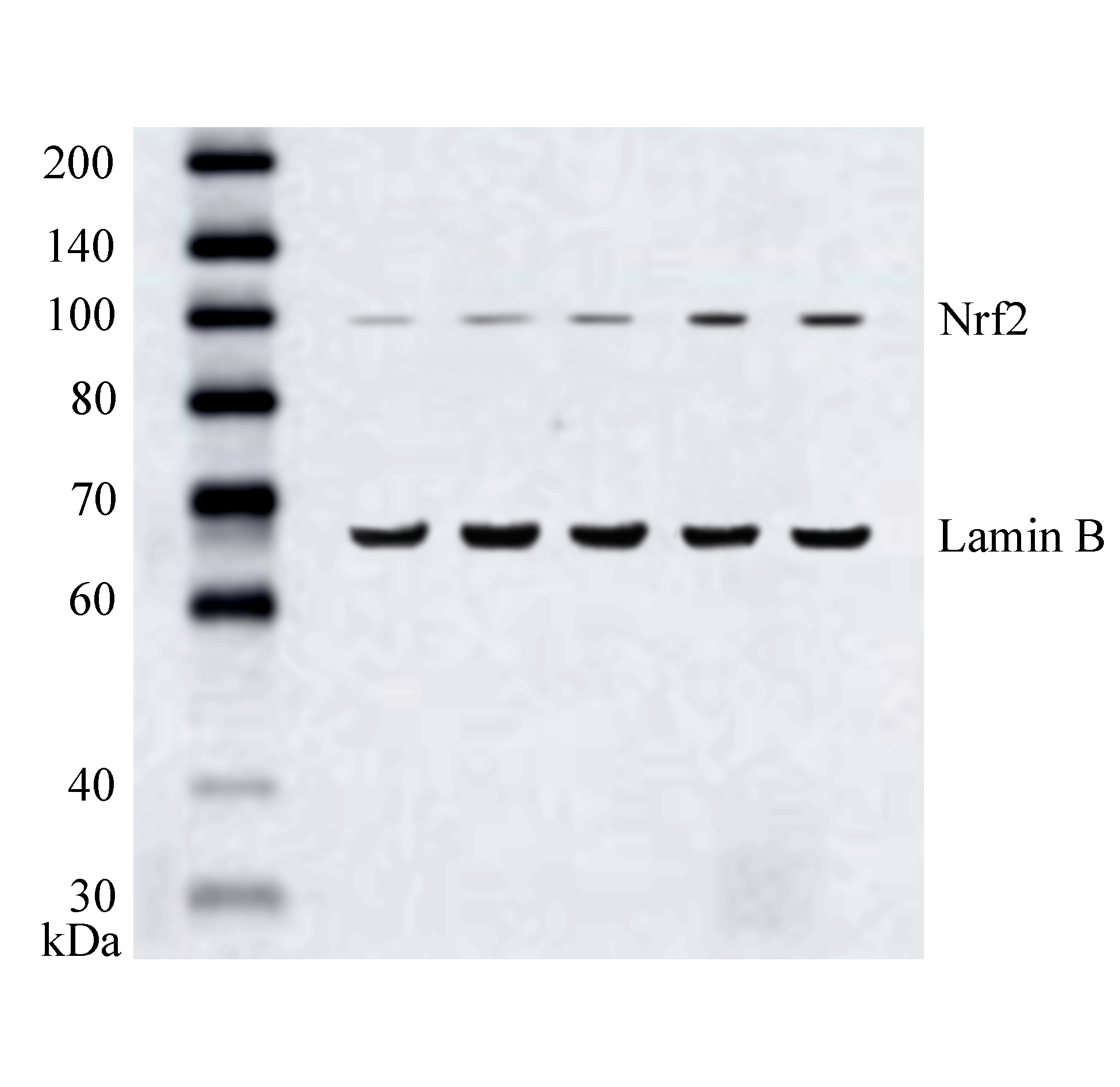

Supplement: S1 Fig — (TIF) [file pone.0245050.s001.tif]

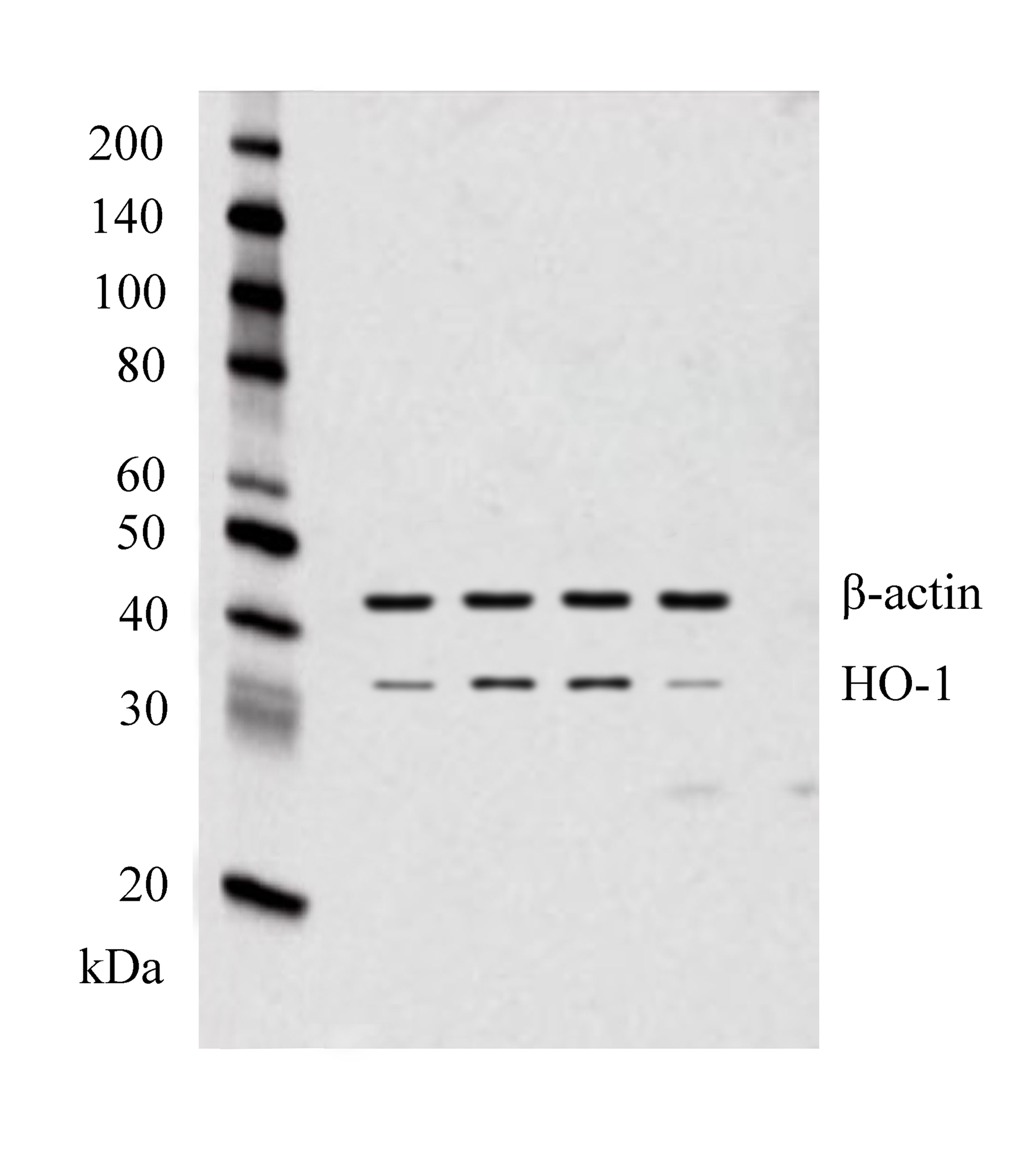

Supplement: S2 Fig — (TIF) [file pone.0245050.s002.tif]
